# Optical coherence tomography findings in three patients with Werner syndrome

**DOI:** 10.1186/s12886-022-02660-z

**Published:** 2022-11-19

**Authors:** Tatsuya Nagai, Hirotaka Yokouchi, Gen Miura, Masaya Koshizaka, Yoshiro Maezawa, Toshiyuki Oshitari, Koutaro Yokote, Takayuki Baba

**Affiliations:** 1 Department of Ophthalmology and Visual Science, Chiba University Graduate School of Medicine, Inohana 1-8-1, Chuo-ku, 260-8670 Chiba, Japan; 2 Department of Endocrinology, Hematology and Gerontology, Chiba University Graduate School of Medicine, Chiba, Japan; 3 Department of Ophthalmology, International University of Health and Welfare School of Medicine, Narita, Japan

**Keywords:** Werner syndrome, Optical coherence tomography (OCT), Retinal thickness, Choroidal thickness, Juvenile cataracts

## Abstract

**Background:**

Werner syndrome is a rare, autosomal recessive disorder characterised by premature aging. It is a typical hereditary progeroid syndrome that can be difficult to diagnose owing to its rarity and the similarity of some of its symptoms, such as juvenile cataracts, to other common ophthalmologic conditions. Early onset of bilateral cataracts is currently used as the ophthalmological feature for Werner syndrome; however, ophthalmologists often find performing a detailed examination of the medical history and genetic testing for Werner syndrome at the time of an ophthalmologic consultation challenging. If a unique ocular finding was observed on ocular examinations in cases of juvenile bilateral cataracts, we could consider Werner syndrome as a differential diagnosis.

**Case presentation:**

We documented the cases of three patients with Werner syndrome in whom thinning of the retina in the retinal nerve fiber layer (RNFL) and ganglion cell complex (GCC) were observed using optical coherence tomography (OCT). Visual field tests revealed the loss of visual field mainly owing to glaucoma.

The thinnig of the choroidal thickness (CT) in three patients was also observed using enhanced depth imaging (EDI)-OCT.

**Conclusions:**

Three patients have thinning of the RNFL, GCC, and choroidal thickness and the loss of visual field. These findings suggest the need for including Werner syndrome in the differential diagnosis when patients presenting with juvenile cataracts of unknown cause also show abnormal retinal and choroidal thinning in the OCT images.

## Background

Werner syndrome is a rare, autosomal recessive disorder characterized by premature aging. The known major signs of Werner syndrome include premature hair changes, bilateral cataracts, atrophic hardening and intractable skin ulcers, soft tissue calcification, bird-like face, and Achilles’ tendon calcification. In addition, atherosclerosis is one of the most important features of this disease. Many patients with Werner syndrome die by their mid-50s owing to diseases such as cancer or arteriosclerosis. Calcification of the Achilles tendon is considered particularly useful for the clinical diagnosis of Werner syndrome owing to its high sensitivity and specificity. The estimated number of patients with Werner syndrome in Japan is approximately 700–2000 [1–4]. In 1992, a causative gene was identified in 8p12 of the short arm of chromosome 8 [5], and in 1996, the gene responsible for this disease, i.e. *WRN*, which encodes RecQ-type DNA helicase, was discovered [6]. In terms of eye-related complications, the positive rate of cataracts is 100% (with a 96% positive rate for cataracts in both eyes) [7]. Certain reports exist of patients with Werner syndrome with cystoid macular edema [8, 9], bilateral retinal detachment [10], or primary bullous keratopathy [11]; however, few reports have documented the changes in the thickness of the retina and choroid. In healthy people, aging causes thinning of the retina and choroid [12, 13] and similar changes are speculated to occur in patients with Werner syndrome. This report documents three cases of Werner syndrome in which thinning of both the retina and the choroid was observed using optical coherence tomography (OCT) (Table 1).

**Table 1:** Laboratory findings in the three patients

		Case 1	Case 2	Case 3
Sex		male	male	female
Age		44	57	47
BMI	(kg/m^2^)	19.4	13.5	19.3
Hematology				
WBC	(/μL)	9,100	7,800	7,100
RBC	(/μL)	404 × 10^4^	339 × 10^4^	415 × 10^4^
Hb	(/dL)	13.5	10.8	11
PLT	(/μL)	161 × 10^3^	326 × 10^3^	387 × 10^3^
Biochemistry				
AST	(U/L)	44	29	19
ALT	(U/L)	52	25	20
ALP	(U/L)	223	298	472
y-GTP	(U/L)	47	47	215
BUN	(mg/dl)	12	22	14
Cre	(mg/dl)	0.81	1.13	0.42
Na	(mmol/L)	138	141	149
K	(mmol/L)	4.5	4.7	4.4

## Case presentation

### Case 1

The case was that of a 44-year-old Japanese male who presented with six major signs of Werner syndrome, including grey hair, cataracts in both eyes, intractable skin ulcers, soft tissue calcification, Achilles’ tendon calcification, and a bird-like face. The condition was attributed to a compound heterozygous mutation of type 4 mutation (c. 3139-1G > C) and novel mutation. His height was 168 cm and weight was 55 kg (body mass index [BMI]: 19.4). He had grey hair and hair loss since his 30s. Moreover, since his 40s, he had right Achilles’ tendon and outer ankle ulcers, skin hardening and atrophy, soft tissue calcification, bird-like face, a high-pitched and hoarse voice, diabetes, dyslipidaemia, osteoporosis, latent hypothyroidism, atrophic thyroiditis, and sarcopenic obesity. At the age of 38 years, he had undergone cataract surgery in both eyes and had been treated with glaucoma eye drops.　He had no family history of Werner syndrome. At the initial visit, his best-corrected visual acuity (BCVA) was 1.2 and 1.2 in the right and left eyes, and the refraction was − 1.00 diopter sphere (DS) / -0.50 diopter cylinder (DC) × 125° and − 0.50 DS / -0.50 DC × 120° in the right and left eye, respectively. The axial length was 23.68 mm and 23.60 mm in the right and left eyes. The patient was already diagnosed with glaucoma and followed up at previous eye clinics before referral to our hospital. The intraocular pressure (IOP) was 6 mmHg in both eyes with treatment using tafluprost, timolol maleate, and brimonidine for both eyes.

**Fig. 1:**
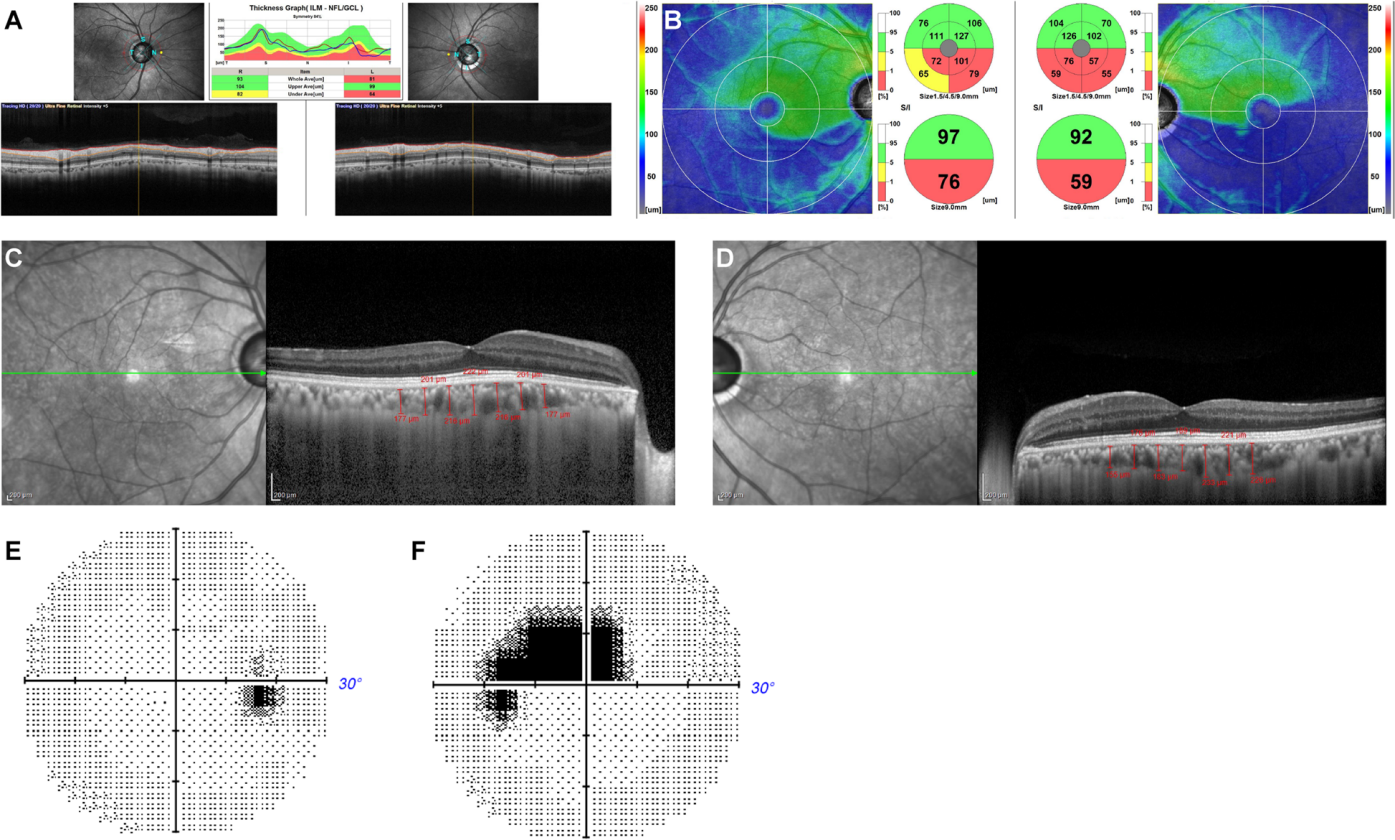
Optical coherence tomography (OCT) images in case 1 showing the circumpapillary retinal nerve fiber layer (cpRNFL) thinning in both eyes (**A**) and the ganglion cell complex (GCC) thinning at the bottom in both eyes (**B**). Enhanced depth image (EDI)-OCT images in case 1 showing choroidal thickness of the right (**C**) and left (**D**) eye. Mean deviation of the Humphrey Field Analyser 30 − 2 test is -0.19 dB and − 5.14 dB in the right (**E**) and left (**F**) eye, respectively. S: Superior, N: Nasal, I: Inferior, T: Temporal, ILM: Inner limiting membrane, NFL: Nerve fiber layer, GCL: Ganglion cell layer

He had intraocular lenses (IOLs) in both eyes. The OCT images were all taken after the cataract surgery. The choroidal thickness was determined from the SD-OCT (Heidelberg Spectralis OCT; Heidelberg, Germany) images. We used enhanced depth imaging (EDI) methods under the same settings. Each image was obtained using the eye tracking system, and 100 scans were averaged to increase the signal-to-noise ratio, and the EDI-OCT algorithm was used. The choroidal thickness was manually measured from the outer border of the retinal pigment epithelium (RPE) to the inner border of the sclera. The RNFL and GCC were automatically measured from the inner layer membrane to the inner border of the RPE with the software in the RS3000 advance spectral domain OCT (SD-OCT; NIDEK, Gamagori, Japan). OCT showed that the average circumpapillary retinal nerve fiber layer (cpRNFL) thickness was 93 μm in the right eye and 81 μm in the left eye, indicating cpRNFL thinning in the left eye (Fig. 1A). Additionally, the ganglion cell complex (GCC) thickness was 97 μm at the superior macula and 76 μm at the inferior macula in the right eye and 92 μm at the superior macula and 59 μm at the inferior macula in the left eye, indicating GCC thinning at the inferior macula in both eyes (Fig. 1B). The central choroidal thickness (CCT) was 222 μm in the right eye (Fig. 1C) and 189 μm in the left eye (Fig. 1D). Mean deviation of the Humphrey Field Analyser 30 − 2 test was − 0.19 dB and − 5.14 dB in the right (Fig. 1E) and left (Fig. 1F) eyes, respectively.

### Case 2

**Fig. 2:**
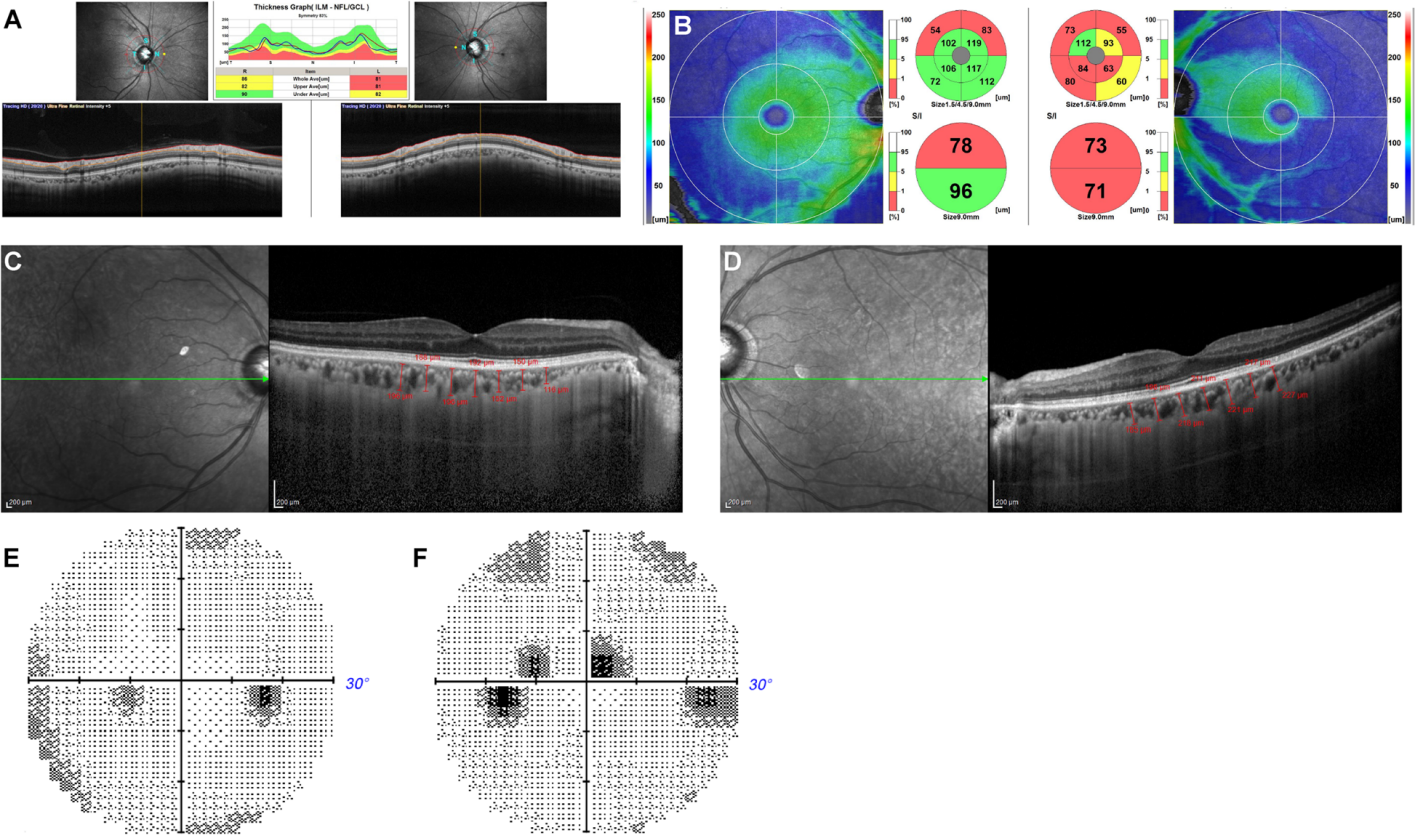
Optical coherence tomography (OCT) images in case 2 showing the circumpapillary retinal nerve fiber layer (cpRNFL) thinning in both eyes (**A**) and the ganglion cell complex (GCC) thinning at the top in both eyes and the bottom in both eyes (**B**). Enhanced depth image (EDI)-OCT images in case 2 showing choroidal thickness of the right (**C**) and left (**D**) eye. Mean deviation of the Humphrey Field Analyser 30 − 2 test is -2.80 dB and − 4.68 dB in the right (**E**) and left (**F**) eye, respectively. S: Superior, N: Nasal, I: Inferior, T: Temporal, ILM: Inner limiting membrane, NFL: Nerve fiber layer, GCL: Ganglion cell layer

The case was a 57-year-old Japanese male who was referred for cataract surgery because of poor vision of his left eye. He presented with six major signs of Werner syndrome, including grey hair, cataracts in both eyes, intractable skin ulcers, soft tissue calcification, Achilles’ tendon calcification, and a bird-like face. The condition was attributed to a type 6 (c. 1105 C > T) homozygous mutation. His height was 163 cm and weight was 36 kg (BMI: 13.5). He had grey hair and hair loss since his 20s and experienced Achilles’ tendon pain bilaterally and skin hardening and atrophy since his 30s. In his 40s, he developed toe ulcers, osteomyelitis, and a high-pitched and coarse voice. After undergoing surgery for bladder cancer at 39 years of age, he had hypertension, diabetes, dyslipidaemia, osteoporosis, and hypothyroidism. At the age of 40, he underwent cataract surgery in his right eye and developed glaucoma; he had also been treated with glaucoma eye drops. At the initial visit, his best-corrected visual acuity was 1.2 and 0.02 and the refraction was + 0.50 DS / -1.00 DC × 90° and − 1.50 DS in the right and left eye, respectively. The axial length was 24.83 mm and 24.82 mm in the right and left eyes. The IOP was 13 mmHg in the right eye and 15 mmHg in the left eye upon treatment with latanoprost and timolol maleate for both eyes. He had an IOL in the right eye, and a cataract in the left eye. After cataract surgery in his left eye, his visual acuity improved to 1.2 and OCT revealed that the cpRNFL had thinned to 86 μm in the right eye and 81 μm in the left eye (Fig. 2A). Moreover, the GCC was 78 μm at the superior macula and 96 μm at the inferior macula in the right eye and 73 μm at the superior macula and 71 μm at the inferior macula in the left eye, indicating GCC thinning at the superior macula in the right eye and at the entire macula in the left eye (Fig. 2B). The CCT was 192 μm in the right eye (Fig. 2C) and 211 μm in the left eye (Fig. 2D). Mean deviation of the Humphrey Field Analyser 30 − 2 test was − 2.80 dB and − 4.68 dB in the right (Fig. 2E) and left (Fig. 2F) eyes, respectively.

### Case 3

**Fig. 3:**
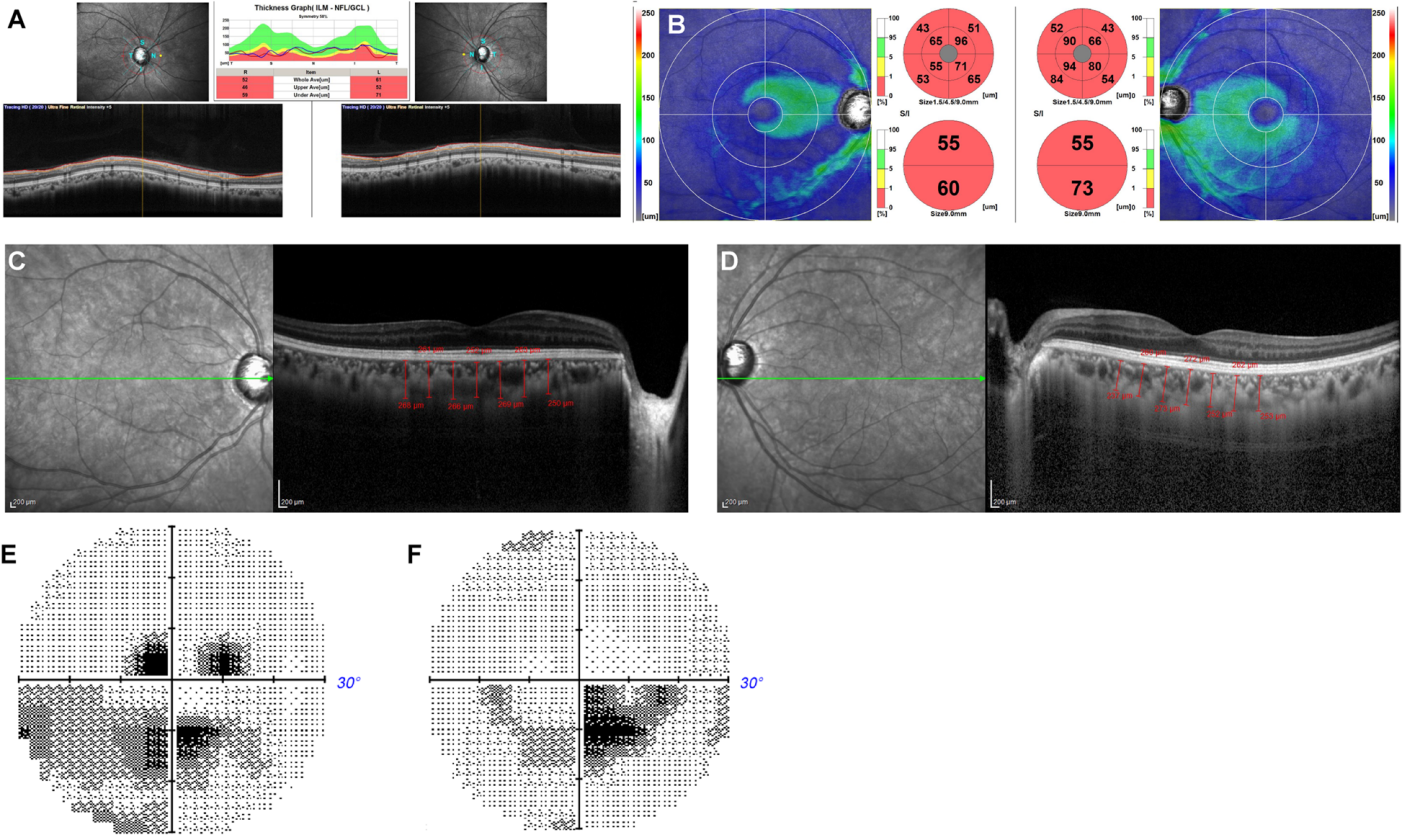
Optical coherence tomography (OCT) images in case 3 showing the circumpapillary retinal nerve fiber layer (cpRNFL) thinning both eyes (**A**) and the ganglion cell complex (GCC) thinning at the top and bottom in both eyes (**B**). Enhanced depth image (EDI)-OCT images in case 3 showing choroidal thickness of the right (**C**) and left (**D**) eye. Mean deviation of the Humphrey Field Analyser 30 − 2 test is -8.47 dB and − 6.52 dB in the right (**E**) and left (**F**) eye, respectively. S: Superior, N: Nasal, I: Inferior, T: Temporal, ILM: Inner limiting membrane, NFL: Nerve fiber layer, GCL: Ganglion cell layer

The case was a 47-year-old Japanese female who presented with five major signs of Werner syndrome, including grey hair, cataracts in both eyes, intractable skin ulcers, Achilles’ tendon calcification, and a bird-like face. The condition was attributed to a compound heterozygous mutation of type 1 (c. 3446delA) and type 4 mutations. Her height was 149 cm and weight was 43 kg (BMI: 19.3). In her 40s, she had developed grey hair, hair loss, hardening of the Achilles tendon on her right leg, ulcers on her left leg, soft tissue calcification, bird-like face, a high-pitched and hoarse voice, diabetes, dyslipidaemia, osteoporosis, and papillary thyroid cancer. At the age of 30, she underwent cataract surgery in both eyes, followed by posterior capsulotomy for secondary cataract in her right eye. At the initial visit, her best-corrected visual acuity was 1.0 and 1.2 and the refraction was + 0.50 and + 0.50 DS / -2.50 DC × 65° in the right and left eye, respectively. The axial length was 24.52 mm and 24.49 mm in the right and left eyes. The IOP was 18 mmHg in both eyes. She had IOLs in both eyes. She showed no signs of diabetic retinopathy. The cpRNFL had thinned to 52 and 61 μm in the right and left eyes while the GCC was 55 μm at the superior macula and 60 μm at the inferior macula in the right eye and 55 μm at the superior macula and 73 μm at the inferior macula in the left eye, indicating GCC thinning at the entire macula in both eyes (Fig. 3A, B). The CCT was 252 μm in the right eye (Fig. 3C) and 272 μm in the left eye (Fig. 3D). Mean deviation of the Humphrey Field Analyser 30 − 2 test is -8.47 dB and − 6.52 dB in the right (Fig. 3E) and left (Fig. 3 F) eye, respectively.

## Discussion and conclusions

Juvenile cataracts frequently occur in the patients with Werner syndrome [14]. In fact, the positive percentage of cataracts is 100% (with a 96% positive percentage for cataracts in both eyes). Therefore, the presence of cataracts is a diagnostic criterion for this disease [7]. Our patients also had the histories of cataract surgery, and two of them already had IOLs in both eyes at their initial visit. Among Japanese people, age-related cataracts are usually observed around the age of 50, with a morbidity less than 10%, and the morbidity increases to 80% or more after the age of 70 [15]. Interestingly, the age of onset of cataracts is extremely young and is usually around 31 years for patients with Werner syndrome [16]. Therefore, most patients consult an ophthalmologist because of poor vision and are diagnosed as having juvenile cataracts of unknown cause at their first visit to an eye clinic. In fact, the causes of juvenile cataracts include diseases that are congenital or atopic dermatitis, as well as diabetes, trauma, and metabolic disorders. Werner syndrome is rarely included in the differential diagnosis because of its rarity.

A national survey in Japan also reported that Werner syndrome was often suggested by dermatologists or plastic surgeons, and not by ophthalmologists [4]. These results suggest that it is necessary to consider measures for early diagnosis and early intervention. The onset of Werner syndrome is usually recognised by bilateral cataracts or grey hair and hair loss, which are usually the first symptoms. However, many of patients and ophthalmologists may not have adequate information to diagnose Werner syndrome. Ophthalmologists often find it difficult to perform a detailed examination of the medical history and genetic testing for Werner syndrome at the time of an ophthalmologic consultation. Therefore, it is difficult for ophthalmologists to make a diagnosis of Werner syndrome.

If a unique ocular finding other than cataracts were observed on ocular examinations in cases of juvenile cataracts in both eyes, we could consider Werner syndrome as a differential diagnosis, and this would lead to an early diagnosis of Werner syndrome. In this report, we documented the cases of three patients with Werner syndrome in whom thinning of the retina and choroid were observed using OCT.

Glaucoma is a well-known disease that causes thinning of the retina owing to the loss of retinal ganglion cells and retinal nerve fiber. The RNFL and GCC start thinning from the early stages of glaucoma, and this finding is useful for its early diagnosis [17]. In addition, aging is considered one of the most important risk factors for the development of glaucoma [18].

The patients in cases 1 and 2 had already been diagnosed with glaucoma and followed up at previous eye clinics before referral to our hospital. And we presume this patient to have glaucoma in left eye upon visual field testing. In fact, Abraham, L.M et al. have also reported primary glaucoma in three siblings with Werner syndrome [19]; however, we could not completely exclude the effect of premature aging as the main feature of this disease.

On the other hand, thinning of the RNFL and GCC also occurs with aging. Leung et al. reported that the cpRNFL thickness naturally decreased by approximately − 0.3 μm per year along with normal aging. An examination showed that the RNFL thickness of the upper area and lower temporal area decreased after the patients reached their 40s [12]. Sung et al. reported that the macular thickness in healthy individuals thinned by approximately − 0.4 μm per year [20], and other studies showed a decrease in the RNFL thickness along with aging [21]. However, the exact cause of this thinning remains unknown.

Therefore, the following question ”Were the changes observed in the cases presented here simply due to the effects of glaucoma?” still remains. We cannot clearly answer this question. Because of that, next, we focussed not only on the retina but also on the choroid in our patients.

The choroid is located between the retina and sclera and is a tissue which is rich in blood vessels and pigments. The choroid can be divided into three layers. The innermost layer is the choriocapillaris, which consists of a dense network of fenestrated capillaries; the second is the Sattler’s layer, which consists of small and medium-sized blood vessels; and the third is the Haller’s layer, which consists of large blood vessels [22]. In healthy individuals, a relationship between choroidal thickness and aging has been reported, and choroidal thickness is known to decrease gradually with aging [23]. Therefore, we focused on the choroidal changes in the OCT images. Considering past literature, the health condition of the patients in Case 1 (44 years old) and Case 2 (57 years old) corresponded to that of a healthy person in their 80s, and the health condition of the patient in Case 3 (47 years old) corresponded to that of a healthy person in their 60s [24]. Other than aging, some factors, such as axial length [25], sex [25], intraocular pressure changes [26], diurnal fluctuations [27], blood glucose levels, and blood pressure treatment [28], have been reported to affect choroidal thickness. However, in our cases, we think that the effect of these factors on choroidal thickness was small, suggesting early age-related changes in the choroid.

Few reports have documented structural retinal changes, especially in the choroid in patients with Werner syndrome, and some cases of complications of glaucoma have also been reported [19]. As described before, the thinning of the cpRNFL and GCC observed on OCT images might be caused not only by an age-related change, but also by a glaucomatous change. However, if we consider choroidal thinning, these retinal and choroidal changes might be due to accelerated aging in patients with Werner syndrome.

When we evaluate age-related retinal and choroidal changes in a healthy human, it is very difficult to follow the changes on OCT images over decades in the same patient. In fact, most of the previous reports on the relationship between aging and choroidal changes are comparative analyses of data measured separately for each age group. However, in Werner syndrome, the same patient can be followed up for several years and we might have the possibility of witnessing the changes similar to those in a healthy human for decades because of the accelerated age-related changes in Werner syndrome. Although more cases and further research are needed in the future, Werner syndrome might be a ‘human retinal/choroidal aging model’ that can help determine aging-related retinal and choroidal changes.

We should describe some limitations of this case report. First, the changes in the choroid and retina might have been due to incidental thinning. Second, these changes might be because of complications, such as glaucoma, which are associated with Werner syndrome. Third, although our cases showed no evidence of diabetic complications, such as diabetic retinopathy, the choroidal and retinal changes could possibly be due to diabetes and the effects of anti-diabetic drugs [29].

Fourth, we could not exclude the effect of atherosclerosis on retinal thickness. It is well known that atherosclerosis, which is an important aspect of Werner syndrome, is associated with reduction of retinal thickness on OCT. For example, previous studies revealed that reduction of retinal thickness is associated with chronic renal failure [30]. No serious atherosclerosis was observed in the fundus of the patients, and they did not have severe renal failure; therefore, we believe that these retinal thickness reductions were not associated with chronic renal failure in our patients.

Finally, the effects of other oral medications, such as blood pressure-lowering drugs, and diurnal fluctuations cannot be excluded.

In our three cases, OCT examination enabled the observation of retinal and choroidal thinning. In order to promote early diagnosis of Werner syndrome, it is necessary to create awareness regarding Werner syndrome among ophthalmologists. When we encounter juvenile cataracts of unknown cause and OCT images show thinning of the retina and choroid unexpected to occur naturally with aging, we should consider Werner syndrome as a differential diagnosis.

## Data Availability

All the data supporting our findings are contained within the manuscript.

## Abbreviations

BCVA: Best-corrected visual acuity
BMI: Body mass index
OCT: Optical coherence tomography
IOP: Intraocular pressure
IOLs: Intraocular lenses
cpRNFL: Circumpapillary retinal nerve fiber layer
GCC: The ganglion cell complex
